# A Mobile Application for Fall Prevention Among Older Adults in Rural Thailand: A Research and Development Approach

**DOI:** 10.34172/hpp.44907

**Published:** 2026-06-06

**Authors:** Chaiyakrit Yokphonchanachai, Yanitha Paengprakhon, Jaruporn Duangsri, Wilawun Chada, Monthicha Raksilp, Nopparat Songserm

**Affiliations:** ^1^Department of Occupational Health and Safety, Faculty of Public Health, Ubon Ratchathani Rajabhat University, Ubon Ratchathani, Thailand; ^2^Department of Community Health, Faculty of Public Health, Ubon Ratchathani Rajabhat University, Ubon Ratchathani, Thailand; ^3^Department of Health Sciences, Faculty of Public Health, Ubon Ratchathani Rajabhat University, Ubon Ratchathani, Thailand

**Keywords:** Fall prevention, Older adults, Mobile application, Caregiver, Digital health, Thailand

## Abstract

**Introduction:**

Falls are a significant cause of injury and reduced quality of life among older adults in Thailand, particularly in rural areas with limited access to preventive care. While several fall prevention programs exist, few are tailored to the caregiver’s role in supporting older adults at home, particularly caregivers in rural settings. This study aimed to develop and evaluate a mobile application for fall prevention among older adults in rural Thailand using a research and development (R&D) approach.

**Methods:**

The five-step R&D process (April 2022–December 2023) included a needs assessment, followed by co-design workshops with caregivers, prototype development and small-group testing, pilot testing for usability and satisfaction, and implementation and evaluation. Through this structured, caregiver-centered process, the application KANLOM was developed. A one-group pretest–posttest study was conducted with thirty caregivers. Outcomes (knowledge, attitudes, and behaviors) were analyzed using descriptive statistics and paired t-tests.

**Results:**

After using the KANLOM application, caregivers demonstrated statistically significant improvements in knowledge (mean difference=1.07, 95% CI: 0.12–2.00, *P*=0.028), attitudes (mean difference=0.13, 95% CI: 0.07–0.23, *P*<0.001), and fall prevention behaviors (mean difference=0.16, 95% CI: 0.08–0.24, *P*<0.001). Overall satisfaction with the development and use of the application was high (mean=2.80 out of 3.00), especially in content usefulness, visual appeal, and practical applicability.

**Conclusion:**

The KANLOM mobile application improved caregivers’ ability to assess and prevent fall risks. Its participatory, offline design supports use in low-resource settings, empowers caregivers to take an active role in fall prevention, promotes community participation and health equity, and provides important implications for future research and policy.

## Introduction

 The global population is aging at an unprecedented rate, posing critical challenges to public health systems worldwide. As of 2020, the global population reached 7.795 billion, with individuals aged 60 and older accounting for approximately 14% (1.05 billion). This number is projected to more than double by 2050, reaching over 2.1 billion, with the fastest growth occurring in low- and middle-income countries.^1^ In the ASEAN region, older adults comprise 11% of the population, with projections indicating that this figure will rise to 31.41% by 2040. Similarly, Thailand is transitioning into an entirely aging society, with those aged 60 and above accounting for over 18% of the population as of 2022.^2^

 Among Thailand’s regions, the northeast has the highest proportion of older adults, with Ubon Ratchathani ranking third in the region and fifth nationally in terms of the size of its older adult population.^2^ Falls are a significant public health concern in this demographic. Approximately one-third of older adults experience at least one fall each year, with increasing incidence among those aged 65 and older.^3^ These falls often result in severe injury, long-term disability, or death.^4^ Intrinsic risk factors include muscle weakness, chronic illness, cognitive decline, and impaired vision. In contrast, extrinsic risks stem from unsafe home environments, such as slippery surfaces and poor lighting.^5^ These risks can diminish an older adult’s quality of life, increase dependence, and lead to psychological impacts such as fear of falling.^3^ Importantly, falls are largely preventable through health promotion strategies involving risk assessment, environmental modification, and behavioral change, with caregivers playing a key role in supporting safer practices at household and community levels.^3^

 While Thailand has implemented fall prevention infrastructure, such as home modifications and community health education, there is limited evidence on mobile applications specifically designed for caregivers in rural areas. Most existing interventions target older adults directly, assuming a level of technological proficiency that many may lack. While some mobile-based fall prevention tools exist,^6^ a key gap remains in interventions that emphasize caregiver empowerment. Most tools target older adults and overlook caregivers, especially in rural and low-resource settings. Inequities in digital access—such as poor connectivity, low digital literacy, and limited technology—also hinder implementation.^7^ These challenges highlight the need for context-specific, caregiver-centered digital solutions.

 Caregivers—particularly family members and village health volunteers (VHVs)—play a crucial role in mitigating daily risks and implementing safety measures for older adults. Despite their central role, caregivers often lack adequate support, training, and tools to perform fall risk assessments and implement appropriate preventive measures. In rural or resource-limited areas, this problem is compounded by limited access to structured digital health innovations that are culturally and contextually appropriate.^7^ These constraints, along with workforce shortages and geographic barriers, limit preventive services and early risk detection, underscoring the need for community-based, digitally supported interventions to promote health equity.^7^

 To address this gap, this study developed a mobile application titled KANLOM, grounded in a participatory research and development (R&D) approach. The application equips caregivers with an accessible, evidence-based tool for assessing and preventing falls among older adults. The specific objectives of the study were as follows: 1) To design and develop a participatory mobile application for caregivers to assess and prevent fall risks among older people; 2) To evaluate the effectiveness of the application in improving caregivers’ knowledge, attitudes, and behaviors related to fall prevention; and 3) To assess caregiver satisfaction and promote application use in high-risk communities in northeastern Thailand.

## Methods

###  Study design

 This research used a five-step R&D framework (April 2022–December 2023) to develop and evaluate the KANLOM mobile application for fall prevention in high-risk older adults: (1) needs assessment; (2) application design and development; (3) multi-phase testing (small group, pilot, field readiness); (4) evaluation and improvement; (5) dissemination. A community-centered approach incorporated input from older adults, caregivers, health professionals, local authorities, and developers to address challenges in rural, resource-limited settings, grounded in public health, geriatric care, and behavioral theory. A participatory approach involved caregivers and stakeholders throughout all phases (needs assessment, co-design, testing, evaluation, implementation, validation, and adaptation) to ensure relevance and user-centered development. The iterative R&D process enabled continuous refinement based on user feedback. The final application aimed to enhance caregiver capacity for fall risk assessment and prevention and was evaluated in a rural setting using a one-group pretest–posttest designdue to practical and ethical constraints.

###  Study setting

**Figure 1:**
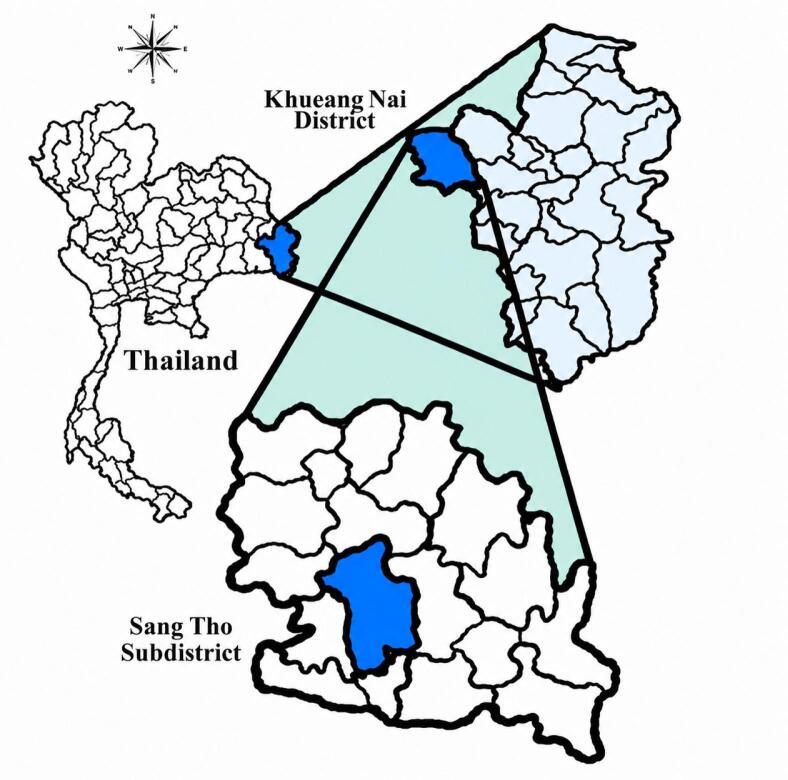
Study area: Sang Tho Subdistrict, Khueang Nai District, Ubon Ratchathani Province—located in northeastern Thailand, approximately 629 kilometers from Bangkok. This rural community served as the implementation site for evaluating the KANLOM mobile application, reflecting typical characteristics of low-resource aging areas in the region.

 The study was conducted in Ubon Ratchathani Province, Northeastern Thailand, an area identified as high-risk for falls among older adults due to its aging population, limited rural infrastructure, and limited fall-prevention resources. The province was selected based on regional health statistics and public health reports highlighting the need for targeted interventions. As illustrated in Figure 1, the primary study site was Sang Tho Subdistrict, Khueang Nai District, where both the needs assessment and field readiness testing were carried out. This subdistrict was chosen for its active older adult school, engaged community health network, and suitability for piloting technology-based interventions in a real-world rural context.

###  Research procedure

**Figure 2:**
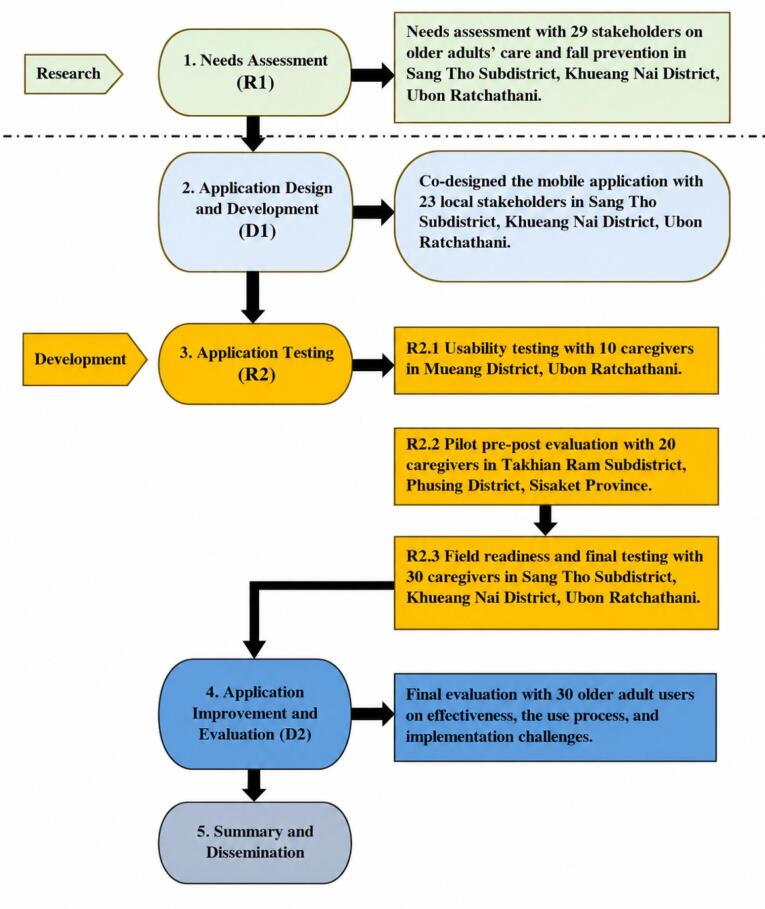
Five-step research and development (R&D) process used to create the KANLOM mobile application for fall prevention among older adults. The steps included: (1) needs assessment through community engagement, (2) co-design and application development, (3) multi-phase testing (small group, pilot, and field implementation), (4) evaluation and refinement, and (5) dissemination. Each step involved caregivers, health professionals, and local stakeholders to ensure contextual relevance, functionality, and cultural appropriateness.

 This study followed a five-step R&D process to develop and evaluate the KANLOM mobile application to prevent falls among older adults (Figure 2). Each phase was grounded in community participation and iterative feedback. A participatory approach at the levels of collaboration and empowerment was applied, engaging caregivers, older adults, health professionals, and community stakeholders across all phases, including needs assessment, co-design, testing, and evaluation, to ensure contextual relevance and user-centered development.

####  1. Needs assessment (R1)

 This initial phase examined the needs, challenges, and contextual factors associated with the care and fall prevention of older adults. A total of 29 stakeholders, including older adults, caregivers, community leaders, local public health officers, and administrative personnel, participated in this step, which was conducted in Sang Tho Subdistrict, Khueang Nai District, Ubon Ratchathani Province. The findings guided the application’s content and design priorities.

####  2. Application design and development (D1)

 In the second phase, 23 stakeholders—comprising software developers, safety academics, geriatricians, public health officers, caregivers, and representatives of older adults—collaborated to co-design the KANLOM application. The design emphasized offline usability, straightforward navigation, and culturally appropriate content tailored to local contexts.

####  3. Application testing (R2)

 This third phase consisted of three sub-steps:

- Small group testing (R2.1): Usability testing was conducted with 10 caregivers in Mueang District, Ubon Ratchathani, focusing on interface navigation and feature accessibility.
- Pilot testing (R2.2): A pre-post evaluation with 20 caregivers in Takhian Ram Subdistrict, Sisaket Province, was conducted to examine initial effectiveness in enhancing fall prevention knowledge, attitudes, and behaviors.
- Field readiness testing (R2.3): The refined application was tested with 30 caregivers in Sang Tho Subdistrict to assess real-world feasibility, satisfaction, and implementation readiness.

####  4. Final evaluation and improvement (D2)

 In this phase, the application was evaluated by 30 older adult users in Sang Tho Subdistrict. The assessment focused on overall effectiveness, user experience, and implementation barriers. Insights from this evaluation informed final improvements prior to broader dissemination.

####  5. Summary and dissemination

 The findings, app prototype, and community feedback were synthesized and prepared for dissemination to relevant stakeholders, with the potential for scaling the intervention in other low-resource settings.

###  Participants and sampling

 Participants were recruited using simple random sampling in each phase to ensure unbiased representation. This was appropriate given the relative homogeneity of caregivers in rural communities. The sampling frame comprised registered caregivers and VHVs identified from community health records, with participants selected using random number generation to ensure equal probability of inclusion.

 Sample sizes were determined separately for each R&D phase according to phase-specific objectives rather than a single rule. In Phase R2.1, 10 caregivers were recruited for formative small-group usability testing. Because this phase was intended to identify usability problems, navigation barriers, and issues of content comprehension in a relatively homogeneous group of intended users, a formal hypothesis-testing power analysis was not applicable. Instead, the sample size was based on methodological guidance for small, focused exploratory testing in tool and intervention development. In Phase R2.2, which used a pre-post design, the sample size was determined for paired comparison analysis. Assuming a two-tailed alpha of 0.05 and 80% power, a sample of 20 participants was sufficient to detect a moderate-to-large within-subject effect (dz = 0.66). In Phase R2.3, 30 caregivers were included to evaluate field readiness and real-world engagement with greater stability; under the same assumptions, this sample could detect a moderate within-subject effect (dz = 0.53). A detailed breakdown of participant groups, settings, and objectives for each phase is presented in Table 1.

**Table 1:** Sampling Framework and Phase-Specific Objectives across the R&D Process for the KANLOM Mobile Application.

**Phase**	**Target Group(s)**	**Location**	**Sample Size**	**Purpose**
R1: Needs assessment	Older adults (n = 5) Caregivers (n = 5) Directors of Subdistrict Health Promoting Hospitals (n = 2) Public health officers (n = 3) School directors of older adults (n = 2) Subdistrict Administrative Organization personnel (n = 5) Community leaders (n = 4) Safety academics (n = 3)	Sang Tho Subdistrict, Khueang Nai District, Ubon Ratchathani Province	29	To explore the needs, problems, and contextual factors related to the care and fall prevention of older adults.
	Older Adults (n = 30)		30	To assess fall risk among older adults using the Morse Fall Scale
D1: Application design and development	Software developers (n = 2) Safety academics (n = 3) Geriatricians (n = 3) Public health officers (n = 2) Caregivers (n = 8) Older adults (n = 5)	Sang Tho Subdistrict, Khueang Nai District, Ubon Ratchathani Province	23	To collaboratively design and develop a mobile application for the safety and fall prevention of older adults.
R2: Application testing (*Small Group Testing*)	Small group of caregivers	Mueang District, Ubon Ratchathani Province	10	To conduct usability testing of the prototype application.
R2: Application testing (*Pilot Testing*)	Pilot group of caregivers	Takhian Ram Subdistrict, Phusing District, Sisaket Province	20	To evaluate the application through a pilot test using a pre-post design.
R2: Application testing (*Field Readiness Testing*)	Sample group of caregivers	Sang Tho Subdistrict, Khueang Nai District, Ubon Ratchathani Province	30	To assess field readiness and conduct a final evaluation prior to full-scale implementation.
D2: Application improvement and evaluation	Older adult users	Sang Tho Subdistrict, Khueang Nai District, Ubon Ratchathani Province	30	To evaluate the effectiveness of the final application, including its processes, outcomes, and challenges encountered.

###  Measurement tools

 The research tools were divided into two primary categories:

####  1. Application development tools (R1, D1, and R2 stages) 

 Includes older adults’ health screening tools (vision, osteoarthritis, Timed Up and Go test (TUGT), and home environment), the Glide App, Google Sheets, and in-depth interview guides.

####  2. Application evaluation tools (R2 and D2 stages) 

 Comprises structured questionnaires assessing five domains:

- *Section 1*: Personal information: 16-item demographic questionnaire.
- *Section 2*: Knowledge of home safety: 15 actual/false questions (KR-20 = 0.88).
- *Section 3*: Attitudes towards home safety: This section was assessed using a 3-point Likert scale (agree, unsure, disagree). This simplified format was chosen to facilitate understanding and reduce cognitive burden among caregivers with varying levels of education, particularly those in rural communities. (IOC = 0.86; α = 0.71). Prior to full implementation, the questionnaire was pilot-tested with 10 caregivers during the small group testing phase (R2.1) to ensure clarity, cultural appropriateness, and comprehension. Feedback from this pilot test led to minor wording adjustments to enhance understanding in the local context. While a 3-point scale offers ease of use and reduces confusion, it may limit response granularity compared to 5- or 7-point scales. However, the research team prioritized accessibility and response reliability over statistical precision in this context. The use of a concise scale ensured higher completion rates and a more accurate reflection of caregivers’ general attitudes.
- *Section 4*: Safety behaviors: Measured with a 3-point frequency scale (always, sometimes, never), appropriate for capturing routine caregiving practices in a practical and respondent-friendly manner. (IOC = 0.92; α = 0.79). This section was also included in the small-group pilot test to determine whether rural caregivers could effectively differentiate between frequency categories. Minor revisions were made to the response instructions to support consistent interpretation.
- *Section 5*: Morse fall risk assessment tool: The Morse Fall Risk Assessment Tool—a widely used 6-item scale designed to evaluate the likelihood of patient falls—was integrated into the KANLOM application as a supplementary reference to enhance caregivers’ understanding of fall risk factors.^8^ In this study, the tool was employed not as an outcome measurement but as an educational aid to guide caregivers’ learning and decision-making processes during the intervention. The inclusion of this standardized tool aimed to increase caregiver awareness and promote early recognition of key risk indicators in older adults. Internal validation conducted by the research team showed high content agreement (IOC = 0.87) and strong internal consistency (α = 0.91). However, scores from the Morse scale were not included in the statistical analysis of program outcomes, as the research prioritized practical applicability and user-centered learning over formal evaluation through this instrument.

###  Statistical analysis

 Data were analyzed using IBM SPSS Statistics for Windows, version 26.0 (IBM Corp., Armonk, NY, USA). Descriptive statistics were used to summarize general information, including frequency distributions, percentages, means, and standard deviations. Prior to hypothesis testing, data distributions for knowledge, attitude, and behavior scores were examined for normality using the Shapiro–Wilk test. The results confirmed normality, allowing the use of parametric methods. Inferential statistics were applied using a paired-samples t-test to test the research hypotheses and to evaluate mean differences before and after the intervention. The paired t-test was chosen due to its appropriateness for analyzing pre- and post-intervention scores within the same group, assuming normality. A statistical significance level of *P* < 0.05 was set. This analysis enabled assessment of changes in participants’ knowledge, attitudes, and behaviors following the application’s implementation.

## Results

 The primary outcomes were changes in caregiver knowledge, attitudes, and fall prevention behaviors, while secondary outcomes included user satisfaction and application usability. Results are presented according to the sequential phases of the R&D process as follows:

###  Needs assessment (R1)

 The initial phase explored local needs, challenges, and contextual risk factors associated with falls among older adults. Data were collected from 29 stakeholders in Sang Tho Subdistrict, including older adults, caregivers, health professionals, community leaders, and local administrators. Key risk factors identified were visual impairments, osteoarthritis, unsafe home environments, and limited caregiver knowledge. The assessment used validated tools (IOC = 0.90; α = 0.84) to guide the design of content and app functionality. Key themes and target groups are summarized in Table 1.

 Among the 30 older adult participants who underwent initial fall risk assessment using the Morse Fall Scale, 50.00% were classified as at risk (score 25–50), 46.67% as high risk (score ≥ 51), and only 3.33% had no fall risk (score 0–24), as shown in Table 2. These findings were used to provide contextual understanding and inform risk profiling during the needs assessment phase. It is important to note that the Morse Fall Scale results were used solely as descriptive support to inform application design and community risk awareness. They were not included in the primary statistical analyses evaluating the intervention’s effectiveness. These results represent baseline fall risk among older adults and are presented separately from caregiver outcome measures.

**Table 2:** Initial Fall Risk Assessment of Older Adults Using the Morse Fall Scale (n = 30).

**Morse Fall Risk Score**	**Number**	**Percentage**	**Interpretation**
0–24	1	3.33	Low risk
25–50	15	50.00	Moderate risk
≥ 51	14	46.67	High risk
Total	30	100.00	

*Note:* The Morse Fall Scale was used to assess baseline fall risk among older adults during the needs assessment phase. These results are presented for descriptive purposes only and were not included in inferential analyses.

###  Application design and development (D1)

**Figure 3:**
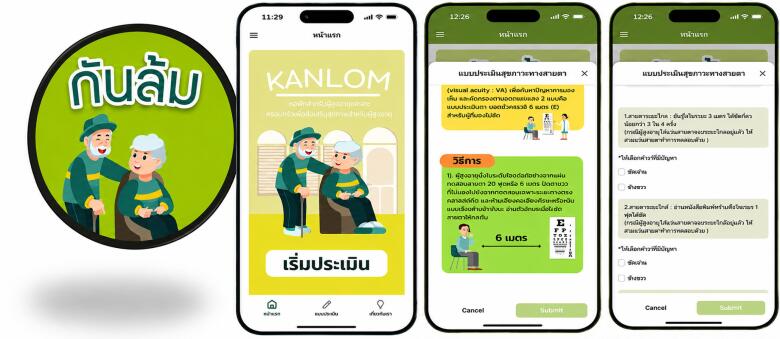
Screenshot of the KANLOM mobile application’s main interface, highlighting core modules and simplified navigation. The design emphasizes usability, visual clarity, and offline functionality. Thai-language labels and localized icons support caregivers in assessing fall risk, implementing preventive measures, and monitoring older adults’ safety, aligned with the intervention domains (knowledge, attitude, and behavior) and components in Table S1.

 A co-design approach was employed to develop the KANLOM application, engaging 23 stakeholxders, including software developers, safety academics, geriatricians, and caregivers. The application was created using Glide App and Google Sheets, featuring tools such as Fall Risk Assessment (e.g., vision, osteoarthritis, TUGT, home safety), Educational content (Knowledge Module), Daily Monitoring checklists, and Offline functionality. Design emphasized user-friendliness and cultural appropriateness to ensure suitability for caregivers in rural settings. The structural linkage between modules and study outcomes is shown in Table S1, and the core application interface is displayed in Figure 3.

###  Application testing (R2): Caregiver outcomes

 This phase presents outcomes measured among caregivers, including knowledge, attitudes, behaviors, and satisfaction. This step consisted of three sub-phases designed to evaluate the usability, satisfaction, and preliminary effectiveness of the KANLOM mobile application among caregivers.

- *R2.1 – Small group testing*: Ten caregivers in Mueang District, Ubon Ratchathani, tested the prototype and provided feedback on usability. The application was rated easy to navigate, accessible offline, and culturally appropriate. Minor revisions were made to icons, sequencing, and instructions.
- *R2.2 – Pilot testing*: Twenty caregivers in Sisaket Province participated in a six-week pilot study, including both pre- and post-evaluation. The results showed increased knowledge, improved attitudes, and enhanced fall prevention behaviors.
- *R2.3 – Field readiness testing*: Thirty caregivers in Sang Tho Subdistrict assessed real-world feasibility and satisfaction. As shown in Table 3, 90% of users reported high satisfaction. Average scores increased over time—2.73 (pre), 2.78 (during), and 2.85 (post)—indicating growing engagement and usability.

**Table 3:** Caregiver-Based Evaluation of the Effectiveness of Each Phase in Developing a Mobile Application for Fall Risk Assessment and Prevention in Sang Tho Subdistrict, Khueang Nai District, Ubon Ratchathani, Thailand.

**Details**	**Satisfaction levels**		
	**Mean**	**SD**	**Interpretation **
Before the implementation	2.73	0.25	High
1. The objectives are clarified.	2.79	0.32	High
2. Contact and coordination with the community are conducted prior to implementation.	2.86	0.21	High
3. The meeting is held, and the goals are set with participation.	2.89	0.19	High
4. The activities are designed for participation.	2.33	0.22	Moderate
5. The plan is implemented with participation.	2.78	0.31	High
During the implementation	2.78	0.22	High
6. The application icon looks interesting.	2.89	0.18	High
7. The name of the application, "KANLOM" (fall prevention), is interesting and easy to remember.	2.79	0.21	High
8. The application is easy to use, convenient, and hassle-free.	2.33	0.19	Moderate
9. The information is appropriate for the assessment.	2.85	0.20	High
10. There is complete information.	2.82	0.15	High
11. The application is attractive to use.	2.93	0.31	High
12. There are helpful suggestions.	2.95	0.25	High
After the implementation	2.85	0.24	High
13. A meeting is held to follow up on activities with participation.	2.87	0.22	Moderate
14. Lessons learned are gathered from every activity following implementation.	2.90	0.21	High
15. Every activity is evaluated.	2.76	0.35	High
16. The results of the activities are communicated to the community.	2.78	0.31	High
17. The results are used to develop and extend knowledge into actual practice.	2.94	0.22	High
18. Communities benefit from the activities.	2.87	0.15	High
Total	2.80	0.23	High

*Note: *Satisfaction scores range from 1.00 to 3.00 and are interpreted as follows: 1.00–1.66 = Low, 1.67–2.33 = Moderate, and 2.34–3.00 = High. Higher mean scores indicate greater satisfaction with the application and participatory activities.

###  Application evaluation and improvement (D2): Older adult outcomes

 This phase presents outcomes for older adults following a caregiver-led assessment using the KANLOM application. This phase involved 30 older adult users (15 males and 15 females; mean age, 64.8 years) from Sang Tho Subdistrict. Most (63.3%) lived with their children and earned an average monthly income of 4,564 THB. Among them, 40% had experienced household accidents, 33.3% had slipped indoors, and 30% had fallen, mainly due to blurred vision (50%). Participants appreciated the application’s interface, offline accessibility, and support for local languages. Minor refinements were implemented based on this feedback.

 The core modules and features of the KANLOM mobile application are detailed in Table S1, which outlines the structure and content corresponding to the key outcome domains: knowledge, attitudes, and fall prevention behaviors. These components were developed based on the needs assessment and refined through co-design and pilot testing to ensure alignment with caregivers’ practical needs in rural settings.

 Statistically significant improvements were observed in older adults after assessment by caregivers using the KANLOM mobile application: knowledge scores rose from 11.83 to 12.90 (mean difference = 1.07, 95% CI: 0.12-2.00, *P* = 0.028), attitudes from 2.58 to 2.71 (mean difference = 0.13, 95% CI: 0.07-0.23, *P* < 0.001), and behaviors from 2.29 to 2.45 (mean difference = 0.16, 95% CI: 0.08-0.24, *P* < 0.001), as detailed in Table 4.

**Table 4:** Changes in Knowledge, Attitudes, and Behaviors Among Older Adults Before and After Assessment by Caregivers Using the KANLOM Mobile Application.

**Variables**	**Before**	**After**	**Mean Difference**	**95% CI**	***P*** **-value**
	**Mean±SD**	**Mean±SD**			
Knowledge	11.83 ± 1.80	12.90 ± 1.40	1.07	0.12 to 2.00	0.028
Attitude	2.58 ± 0.13	2.71 ± 0.15	0.13	0.07 to 0.23	< 0.001
Behavior	2.29 ± 0.15	2.45 ± 0.16	0.16	0.08 to 0.24	< 0.001

*Note: *Statistical significance set at *P* < 0.01. Higher scores indicate better outcomes in caregiver knowledge (maximum = 15), attitudes (range = 1–3), and safety behaviors (range = 1–3).

 The caregiver-performed assessment using the KANLOM mobile application revealed that 53.33% (95% CI: 34.30–71.70) of older adults had high vision risk, and 66.67% (95% CI: 47.20–82.70) were at high risk in their home environment. In the osteoarthritis domain, 33.33% (95% CI: 17.30–52.80) were at high risk, while 56.67% (95% CI: 37.40–74.50) were at low risk. Regarding fall risk, 46.67% (95% CI: 28.30–65.70) were at risk, and 53.33% (95% CI: 34.30–71.70) were not. These findings emphasize the need for targeted interventions, particularly in visual health and household safety (Table 5).

**Table 5:** Caregiver-Performed Risk Assessment of Older Adults Using the KANLOM Mobile Application (n = 30).

**Parameter**	**Risk Level Criteria**	**n**	**%**	**95% CI**	**Interpretation**
Vision	Low ( < 10%) Moderate (10–50%) High (51–100%)	9 5 16	30.00 16.67 53.33	14.70–49.40 5.60–34.70 34.30–71.70	Monitor Improvement recommended Immediate improvement required
Osteoarthritis (Knees)	Low ( < 10%) Moderate (10–50%) High (51–100%)	17 3 10	56.67 10.00 33.33	37.40–74.50 2.10–26.50 17.30–52.80	Monitor Improvement recommended Immediate improvement required
Fall Risk (TUGT)	No risk (0%) At risk (1–100%)	16 14	53.33 46.67	34.30–71.70 28.30–65.70	No risk At risk
Home Environment	Low ( < 10%) Moderate (10–50%) High (51–100%)	3 7 20	10.00 23.33 66.67	2.10–26.50 9.90–42.30 47.20–82.70	Monitor Improvement recommended Immediate improvement required

*Note:* Risk levels were classified as low ( < 10%), moderate (10–50%), and high (51–100%) based on the proportion of identified conditions. Fall risk was assessed using the Timed Up and Go Test (TUGT) and categorized as no risk (0%) or at risk ( ≥ 1%). Abbreviations: CI, confidence interval; TUGT, Timed Up and Go Test; n, number of participants; %, percentage.

###  Summary and dissemination

 The finalized KANLOM application and research findings were disseminated to local health offices and community stakeholders to support future use and policy integration. A logic model (Table S2) was developed to map the pathway from inputs to outcomes. Application screenshots (Figure 3) and module linkages (Table S1) illustrated the practical structure and scalability for replication.

## Discussion

 This study demonstrated that the KANLOM mobile application—developed through the R&D process—effectively improved caregivers’ knowledge, attitudes, and safety behaviors related to fall prevention among older adults. Each step in the R&D process—needs assessment, co-design, small-group testing, pilot testing, and final field implementation—contributed to ensuring the application’s contextual relevance and functional robustness. The participatory, community-based approach was efficient in rural Thai settings, where digital infrastructure and health promotion resources remain limited.

 The success of the intervention reflects growing evidence that mobile health tools can complement, or even surpass, traditional methods in health promotion, highlighting the potential of mobile applications to assess fall risk with comparable reliability to conventional assessments.^6^ Notably, while most fall-prevention tools have focused on older adults themselves, the caregiver-centered model of KANLOM addresses a neglected component in Thailand’s fall-prevention strategies.

 The participatory process was central to the tool’s impact. As noted by Yokphonchanachai et al. ^9^ and Syed et al.,^10^ community engagement in intervention development increases user satisfaction, promotes relevance, and enhances long-term outcomes. The high level of caregiver satisfaction and engagement observed in this study supports these findings and underscores the importance of collaborative design in fostering application acceptance.

 Three core design features likely contributed to the observed improvements in caregivers’ behavior and attitudes. First, the application’s step-by-step instructional layout helped users gradually build confidence in applying fall prevention strategies. Second, offline functionality allowed uninterrupted access to tools and content in rural areas where internet connectivity is often limited. Third, integrating culturally appropriate visuals and local language made the application more accessible to caregivers with diverse educational backgrounds. From a health promotion perspective, however, these features should be understood as enabling tools rather than direct mechanisms of change. The intervention’s effectiveness is better explained by underlying processes, including enhanced caregiver self-efficacy, increased control over the home environment, and reinforcement of safety norms. The application facilitated these processes by translating knowledge into actionable practices, rather than serving as an end in itself. Similar findings have been reported by Turnbull et al.,^11^ whose “SmartCaregivers 1.0” improved perceived usefulness and ease of use, highlighting the value of context-sensitive, participatory mobile design.

 The observed improvements in caregiver competencies support previous evidence that mobile interventions can drive measurable change. For example, a video-based mobile app has significantly enhanced health-related knowledge and decision-making among adults aged 65 and above, with improved eHealth literacy and self-efficacy.^12,13^ Similarly, a systematic review highlighted that structured mobile fall-prevention programs for older adults achieved high adherence (pooled adherence rate, 0.82; 95% CI, 0.68–0.93) and effectively supported balance and fall-prevention skills.^14^

 Beyond individual-level improvements, the KANLOM application also presents potential community-level benefits. By equipping caregivers with tools for early risk detection and preventive action, the application may help reduce the incidence of fall-related injuries among older adults. Evidence suggests that mobile health (mHealth) interventions can effectively reduce falls in older populations, particularly in low- and middle-income settings where access to conventional healthcare services is limited.^15^ This proactive approach may lower the burden on local healthcare systems and reduce long-term treatment costs for families and public health authorities. Moreover, cost-effectiveness evaluations—such as the study of the “Safe Step” digital intervention—have demonstrated that fall prevention interventions can yield measurable savings and improved health outcomes compared to no intervention.^16^ These findings support the scalability and policy relevance of caregiver-centered mobile applications, such as KANLOM, particularly in rural and resource-constrained areas. The tool aligns with broader goals of promoting health equity and aging-in-place strategies in rapidly aging societies such as Thailand.

 The use of a simplified 3-point Likert scale, while suitable for caregivers with limited education, may have restricted the ability to detect subtle changes in responses. Previous psychometric studies indicate that using more granular scales (e.g., 5-point or 7-point) can offer greater sensitivity and reliability, reducing central-tendency bias and capturing nuanced shifts more effectively.^17^ Nonetheless, prioritizing accessibility and minimizing respondent fatigue was appropriate for the target setting.

 Structurally, the KANLOM mobile application was intentionally designed to align with caregivers’ needs and the study’s outcomes. Its modular design included a fall risk assessment tool, educational content, daily monitoring functions, and a support-and-feedback interface, developed through co-design with end-users to ensure relevance and usability in real-world settings. Such co-design approaches, as demonstrated in mHealth tools for dementia caregivers, utilize needs assessment phases to develop distinct content modules.^18,19^ Moreover, systematic reviews of mobile fall-risk assessment applications confirm that modular features, such as balance evaluation, can be implemented with acceptable validity and reliability, thereby reinforcing the internal consistency of intervention components.^20,21^

 In summary, KANLOM provides an accessible, community-grounded tool to empower caregivers in preventing falls among older adults. Its success in rural Thailand points to broader applicability in similar low-resource contexts. Future efforts should focus on exploring longer-term outcomes, scalability, and adaptation for other caregiving populations, such as home-based aides and institutional care workers.

## Limitations

 This study has some limitations. First, the small sample sizes in each R&D phase, despite using purposive and random sampling, may limit the generalizability of the findings beyond the study context. Replication in diverse settings is needed to enhance external validity. Second, the quasi-experimental design, lacking a control group, limits causal interpretation. Third, the Morse Fall Risk Assessment Tool was used for reference purposes only and was not included in the outcome analysis. Lastly, only short-term outcomes were assessed; long-term effects and sustained behavior change remain unknown.

## Conclusion

 The KANLOM mobile application significantly enhanced caregivers’ capacity to assess and prevent falls among older adults in high-risk rural areas of Thailand. Developed through an R&D approach, the application demonstrated high usability, intense satisfaction, and measurable improvements in caregiver knowledge, attitudes, and safety behaviors. The results support the effectiveness of community-based, co-designed digital tools for caregiver education and fall prevention. Given its offline functionality, localized content, and simple design, the application is well-suited for adoption in other low-resource, aging communities.

## Recommendations

1. Conduct follow-up research over 6–12 months to assess the sustainability of behavioral change and potential reductions in fall incidence among older adults.
2. Scale up the application in similar rural settings, given its high satisfaction and offline usability.
3. Pilot the application with other caregiver groups (e.g., home-based aides, institutional staff) to test broader applicability.
4. Integrate the application into community health services, supported by training for caregivers and VHVs.
5. Develop multilingual or culturally adapted versions for regional use in aging ASEAN populations.

## Competing Interests

 There was no conflict of interest.

## Ethical Approval

 This study was approved by the Human Research Ethics Committee of Ubon Ratchathani Rajabhat University (Ref. No. HE662064). Verbal informed consent was obtained from all participants prior to their involvement in the study. This approach was deemed appropriate and approved by the ethics committee due to the characteristics of the participants, who were primarily caregivers in rural areas with limited literacy. All participants were fully informed of the study’s objectives, procedures, potential risks, and their rights to withdraw at any time without consequence.

## Supplementary Files

Supplementary file contains Table S1 and Table S2.
