# Supplementary material for: A Mobile Application for Fall Prevention Among Older Adults in Rural Thailand: A Research and Development Approach
Source: Health Promot Perspect. 2026 Jun 6;16(1):111–20. doi: 10.34172/hpp.44907 (PMC13402666; doi:10.34172/hpp.44907)
Supplement: Supplementary file 1 — Supplementary file contains Table S1 and Table S2. [file hpp-16-111-s001.pdf]

Yokphonchanachai et al, Health Promotion Perspectives 2026;16(1):111-S1
doi:10.34172/hpp.44907
https://hpp.tbzmed.ac.ir

Supplementary Files

Table S1. The structure of the KANLOM mobile application outlines its main functional modules, corresponding features, and their linkage to the study's outcome indicators. This structure illustrates how the application was designed to support caregivers in improving knowledge, attitudes, and safety behaviors related to fall prevention among older adults.

Application Module	Functions / Features	Linked Outcome Domains	Example Indicators / Outputs
1. Fall Risk Assessment	Includes TUGT, vision screening, osteoarthritis checklists, and environmental safety assessments	Knowledge of fall risks and environmental hazards	TUGT score awareness, identification of fall hazards at home
2. Knowledge Module	Provides educational content, illustrated instructions, and key messages for caregivers	Attitudes toward fall prevention and safety practices	Increased understanding of fall causes and prevention tips
3. Daily Monitoring	Offers daily checklists and routine monitoring tools for caregivers	Safety behaviors in routine caregiving	Use of daily checklists, improved routine safety behaviors
4. Support and Feedback	Enables users to log observations, report issues, and access help resources	User satisfaction and sustained engagement	Help requests submitted, engagement with support content

Table S2. The logic model for the KANLOM mobile application intervention. It shows the logical pathway from inputs and activities to outputs and short-term outcomes, demonstrating how the intervention is designed to influence caregiver knowledge, attitudes, and behaviors regarding fall prevention.

Inputs	Activities	Outputs	Short-Term Outcomes
• Research team<br>• Public health officers<br>• Application developers<br>• Community resources	• Needs assessment<br>• Application design & development<br>• Caregiver training<br>• Multi-phase testing	• Mobile application prototype<br>• Educational modules<br>• Risk assessment tools<br>• Feedback systems	• Improved caregiver knowledge<br>• Greater safety awareness<br>• Fall prevention behaviors
